# Screening of Highly Virulent *Beauveria bassiana* Strains Against *Tuta absoluta* Larvae and Evaluation of Their Endophytic Colonization-Mediated Suppression in Tomato Plants

**DOI:** 10.3390/plants14182932

**Published:** 2025-09-21

**Authors:** Bo Xu, Cong Huang, Sheng Cheng, Jörg Romeis, Jana Collatz, Guifen Zhang, Yibo Zhang, Guohui Zhang, Fanghao Wan

**Affiliations:** 1 Engineering Research Center of Ecology and Agricultural Use of Wetland, Ministry of Education, Hubei Key Laboratory of Waterlogging Disaster and Agricultural Use of Wetland, College of Agriculture, Yangtze University, Jingzhou 434025, China; xubo_insect01@163.com (B.X.);; 2 State Key Laboratory for Biology of Plant Diseases and Insect Pests, Key Laboratory for Prevention and Control of Invasive Alien Species of Ministry of Agriculture and Rural Affairs, Institute of Plant Protection, Chinese Academy of Agricultural Sciences, Beijing 100193, China; 3 Agroscope, Research Division Agroecology and Environment, Reckenholzstrasse 191, 8046 Zurich, Switzerland

**Keywords:** *Beauveria bassiana*, *Tuta absoluta*, tomato, endophytes, biocontrol

## Abstract

To identify highly virulent *Beauveria bassiana* strains against *Tuta absoluta* and evaluate their biocontrol potential, four strains were phylogenetically characterized via ITS sequence analysis of rDNA and assessed for virulence against second-instar *T. absoluta* larvae. Foliar spray and root irrigation methods were used to establish *B. bassiana* endophytic colonization in tomato plants, with untreated plants serving as controls. A population life table was constructed to quantify the impact of colonized plants on larval development, fecundity, and key demographic parameters. Results showed variation in virulence among the four *B. bassiana* strains Bb1Bm, Bb2Bm, Bb1M, and BbC with Bb1Bm exhibiting the highest pathogenicity (85.00% corrected mortality at 1 × 10^8^ spores/mL). Maximum endophytic colonization in tomato leaves was observed 14 days post-inoculation with both foliar spray and root irrigation treatments. Life table analyses revealed that *T. absoluta* feeding on colonized plants exhibited significantly reduced survival rates, shorter adult lifespans, and lower female fecundity compared to controls. Key population parameters, including net reproductive rate (*R*_0_), intrinsic rate of increase (*r*), and finite rate of increase (*λ*), were significantly reduced, while mean generation time (*T*) was significantly prolonged. These findings highlight the dual role of *B. bassiana* in *T. absoluta* management, demonstrating its potential as both a direct pathogen and an endophytic biocontrol agent capable of disrupting pest population dynamics.

## 1. Introduction

Tomato (*Solanum lycopersicum*) is one of the world’s most economically significant vegetable crop, with China leading global production and continuously expanding cultivation areas [1]. The South American tomato pinworm, *Tuta absoluta* (syn: *Phthorimaea absoluta*) (Lepidoptera: Gelechiidae), is a major pest of tomato crops, with larvae acting as leaf miners that tunnel into leaves, stems, and terminal buds, causing extensive necrosis [2]. Feeding within the mesophyll tissue reduces photosynthetic capacity and significantly diminishes yields [3]. Without effective management, *T. absoluta* infestations can lead to 80–100% annual yield losses [4].

Chemical insecticides have been widely used to control *T. absoluta* [2]. However, continuous pesticide applications have led to rapid resistance development in *T. absoluta* populations [5,6], diminishing their efficacy. Additionally, excessive pesticide use disrupts natural enemy populations and contributes to environmental contamination [7,8,9]. Given these challenges, sustainable and environmentally friendly pest management strategies are urgently needed. Biopesticides, particularly entomopathogenic fungi (EPF), have emerged as promising alternatives and are increasingly integrated into pest management programs [10,11].

Biocontrol agents, such as entomopathogenic fungi, have prominent virulence factors and have rapidly emerged as prime substitutes for synthetic insecticides [12]. EPF, such as *Beauveria bassiana*, offer an effective and environmentally friendly approach to pest suppression [13]. *B. bassiana* has been successfully established as an endophyte in multiple crop species, including tomato [14], tobacco [15], grape [16], and cowpea [17]. As an endophyte, *B. bassiana* not only directly kills insect pests but also enhances plant defense responses, reducing herbivore damage [16,18]. Additionally, it produces specialized metabolites that exert insecticidal effects beyond the fungal infection [19]. Among lepidopteran pests, *B. bassiana* has demonstrated high virulence, making it a strong candidate for *T. absoluta* management [20].

Research on screening highly virulent *B. bassiana* strains for *T. absoluta* control has gained global attention [21,22]. In Ethiopia [23] and Tunisia [24], researchers have identified *B. bassiana* strains with strong pathogenicity against *T. absoluta*, while commercial formulations are being evaluated for large-scale implementation [25,26,27]. Studies also indicate that *B. bassiana* endophytic colonization in tomato plants enhances its effectiveness against *T. absoluta*. For instance, tomato plants colonized by *B. bassiana* strain LPP139 achieved 100% larval mortality [14], while strain SN182 significantly increased larval mortality and reduced leaf damage [28]. These findings suggest that endophytic colonization can effectively suppress *T. absoluta* populations. However, detailed studies on how *B. bassiana* colonization affects *T. absoluta* life history traits and population dynamics remain limited. In particular, research on high-virulence *B. bassiana* strains for *T. absoluta* control in China is still in its early stages.

This study aims to identify a highly virulent *B. bassiana* strain against *T. absoluta* larvae and assess its impact on pest population dynamics when established as an endophyte in tomato plants. By analyzing life table parameters, we seek to clarify the effects of *B. bassiana* colonization on *T. absoluta* development and reproduction, providing a foundation for integrating *B. bassiana* into sustainable pest management strategies.

## 2. Materials and Methods

### 2.1. Entomopathogenic Fungi, Insects, and Plants

Four *B*. *bassiana* strains (Bb1Bm, Bb2Bm, BbC, and BbM) used in this study were isolated from infected *Bombyx mori* (silkworms) purchased from markets, and were provided by Professor Yi Zhou (College of Agriculture, Yangtze University). Although molecular identification was not performed, all strains were purified and maintained under laboratory conditions.

*T. absoluta* larvae were collected in January 2024 from unmanaged tomato greenhouses in Datong City, Shanxi Province, China. The collected colony was maintained in the laboratory on tomato plants (“Fenjiaren” tomato, provided by Shandong Shouhe Seed Industry Co., Ltd., Jinan, China) under controlled conditions (26 ± 1 °C, 75 ± 5% RH, 14:10 h L:D photoperiod). The colony had been reared for two generations prior to the experiment.

Tomato seeds (Zhongza No. 9) were obtained from China Vegetable Seed Technology (Beijing, China) Co., Ltd. Before sowing, seeds were surface-sterilized in 1% NaClO for 5 min with agitation, rinsed three times in sterile distilled water, and dried on sterile filter paper. The growth substrate (nutrient soil/vermiculite = 2.5:1; Beijing Xinbo Weiye Flower Co., Ltd., Beijing, China) was autoclaved at 121 °C for 20 min and cooled to room temperature before use. Sterilized seeds were soaked in sterile ddH_2_O for 2 h, placed on moistened filter paper for germination. After 2 days, seeds were transferred with a sterilized brush to a 64-cell seedling tray containing sterilized substrate (one seed per cell) and grown in an insect-free greenhouse (26 ± 1 °C, 75 ± 5% RH, 14:10 h L:D photoperiod). Seedlings at the two-true-leaf stage were moved to pots (10 cm height × 7.5 cm diameter) filled with sterile substrate and maintained with uniform water and nutrient management until required for experimentation.

### 2.2. Identification of B. bassiana

The four *B. bassiana* strains were cultured on PDA at 26 ± 1 °C in complete darkness for 14 days. Conidia were harvested and submitted to Rui Biotech Co., Ltd., Beijing, China for internal transcribed spacer (ITS) sequencing. The obtained sequences were analyzed using the NCBI BLAST+ 2.17.0 tool to confirm species identity. To clarify phylogenetic relationships, ITS sequences of 14 reported *Beauveria* species were retrieved from the GenBank database (http://www.ncbi.nlm.nih.gov/) (accessed on 28 January 2024), including *Beauveria majiangensis* (NR_158356.1), *Beauveria asiatica* (NR_111596.1), *Beauveria australis* (NR_111597.1), *Beauveria brongniartii* (NR_111595.1), *Beauveria pseudobassiana* (NR_111598.1), *Beauveria amorpha* (NR_111601.1), *Beauveria caledonica* (NR_077147.1), *Beauveria vermiconia* (NR_151832.1), *Beauveria kipukae* (NR_111600.1), *Beauveria sungii* (NR_111602.1), *Beauveria malawiensis* (NR_136979.1), *Beauveria varroae* (NR_111599.1), *Beauveria lii* (NR_111678.1), and *Beauveria bassiana* (ON796013.1). *Cordyceps ningxiaensis* (Cordycipitaceae) (NR_137117.1) was selected as the outgroup. Phylogenetic analysis was conducted using MEGA 7.0 with the neighbor-joining method, and bootstrap support was assessed using 1000 replicates.

### 2.3. Pathogenicity Bioassay of B. bassiana Strains Against T. absoluta

Fungi were cultured as described in Section 2.2. Conidia were harvested, suspended in sterile 0.1% (*v*/*v*) Tween-80 solution, and homogenized by magnetic stirring for 5 min. The suspension was adjusted to 1 × 10^7^ spores/mL using a hemocytometer.

When the transplanted tomato plants described in Section 2.1 reached approximately 35 cm in height, pinnately compound leaves of about 10 cm in length were excised for use in the experiments. Petioles were inserted into 1.5 mL centrifuge tubes filled with water to maintain leaf turgor, and each leaf was placed in a sterile 15 cm Petri dish lined with sterile filter paper. Uniformly sized second-instar *T*. *absoluta* larvae selected from rearing cages were immersed in 100 mL of conidial suspension for 10 s at 21 April 2024. Control larvae were treated with sterile water containing 0.1% (*v*/*v*) Tween-80. Treated larvae were moved onto tomato leaves and incubated under controlled conditions (26 ± 1 °C, 75 ± 5% RH, 14:10 h L:D photoperiod) for 14 days. Mortality was recorded daily, and corrected mortality was calculated using the following formula [29]:

Corrected mortality rate = [(mortality rate of treatment − mortality rate of control)/(1 − mortality rate of control)] × 100%

### 2.4. Lethal Concentration and Time of B. bassiana Bb1Bm Against T. absoluta

The most virulent strain, Bb1Bm (identified in Section 2.3), was evaluated for median lethal concentration (LC_50_) and median lethal time (LT_50_) against *T. absoluta* larvae following Silva et al., (2020) [14]. Spore suspensions at 1 × 10^8^, 1 × 10^7^, 1 × 10^6^, 1 × 10^5^, and 1 × 10^4^ spores/mL were prepared as described in Section 2.2, and uniformly staged second-instar larvae were exposed to each concentration at 10 May 2024. Cumulative mortality was recorded daily for 14 days, and the final mortality rates were analyzed by probit regression. Each concentration treatment was replicated eight times.

### 2.5. Inoculation and Detection of B. bassiana Colonization

A conidial suspension of *B. bassiana* strain Bb1Bm was prepared at 1 × 10^8^ conidia/mL as described in Section 2.3 and used immediately for inoculation. Inoculation was performed when the transplanted tomato plants described in Section 2.1 had developed four pinnately compound leaves. Two inoculation methods were employed: foliar spraying (BbFS) and root irrigation (BbRI).

For the BbFS method, to prevent cross-contamination from soil splash, the lower stem and soil surface were wrapped in cling film before inoculation. A handheld sprayer was used to evenly apply 10 mL of the spore suspension per plant onto the upper leaf surfaces. For the BbRI method, 10 mL of the spore suspension was slowly introduced into the topsoil (0–5 cm depth) surrounding the root zone using a 10 mL syringe. Control plants were sprayed with a 10 mL 0.1% (*v*/*v*) Tween-80 solution on 13 May 2024. All treated plants were maintained at 26 ± 1 °C and 75 ± 5% relative humidity under a 14 h light/10 h dark photoperiod.

Endophytic colonization of *B. bassiana* in tomato tissues was confirmed using PDA isolation. At 3, 7, 14, and 21 days post-inoculation, six plants per treatment were randomly selected. Stems and leaves were excised, triple-rinsed with sterile water to remove epiphytic fungi, and surface-sterilized sequentially in 70% ethanol (1 min), 2% sodium hypochlorite (2 min), followed by three rinses with sterile water. Residual moisture was blotted with sterile filter paper. To verify sterilization effectiveness, negative control plants (non-inoculated) were sprayed with the spore suspension (1 × 10^8^ conidia/mL) and immediately subjected to the same sterilization process before plating.

Sterilized tissue samples were cut into 1 × 1 cm fragments, with 10 fragments per stem/leaf sample plated onto PDA (10 plates per plant). Plates were incubated at 26 ± 1 °C in complete darkness for five days and removed daily at 17:00 for fungal growth monitoring under light conditions. Colonization was recorded as positive if characteristic B. bassiana colonies emerged on at least one plate per plant. The colonization rate (%) was calculated as follows: Colonization rate = (Number of fungal-positive plants/Total plants tested) × 100%.

### 2.6. Age-Stage, Two-Sex Life Table Analysis of T. absoluta Populations Grown on B. bassiana Colonized Tomato Plants

Fifty mated females were confined in mesh cages (50 × 50 × 50 cm) from 20:00 to 24:00 under dark-phase conditions at 10 June 2024, with a tomato plant provided for oviposition. Upon completion of oviposition, the plant bearing eggs was removed, and freshly laid eggs were collected. At 08:00 the following day, 300 eggs were randomly assigned to three treatment groups: (1) BbFS, (2) BbRI, and (3) untreated control, with 100 eggs per group.

Pinnately compound leaves (five primary leaflets) from tomato plants subjected to the corresponding treatments were excised using sterilized scissors and placed in 15 cm Petri dishes with moistened cotton to maintain humidity. One egg was placed on each leaflet, totaling 100 eggs per treatment. Egg and larval development and survival were observed daily at 20:00. Moisture was replenished as needed, and foliage was replaced daily to ensure sufficient feeding.

Upon pupation, pupal sex was determined morphologically, and pupal duration was recorded. Newly emerged adults were paired in a 1:1 ratio within cylindrical plastic chambers (20 cm height × 10 cm diameter) featuring a 1 cm basal aperture. A transparent plastic bowl filled with water was positioned beneath the aperture, with a tomato leaf (from identically treated plants) inserted through the opening so that its petiole remained submerged, preserving turgor for oviposition. Paired adults were provided fresh leaves daily, and fecundity (egg counts) and adult survival were recorded until all individuals had died.

### 2.7. Life Table Analysis

The life table parameters of *T. absoluta* were analyzed using the age-stage, two-sex life table method described by Chi, implemented in TWOSEX-MSChart [30]. Age-stage life expectancy (*e_xj_*), reproductive value (*v_xj_*), and intrinsic rate of increase (*r*) were calculated as per Chi and Liu [31]. The age-specific survival rate (*l_x_*) was calculated as follows: $l_{x}=\sum_{j=0}^{m}s_{xj}$. Age-stage specific fecundity (*f_xj_*) and age-specific fecundity (*m_x_*) were determined using: $m_{x}=\frac{\sum_{j=1}^{m}s_{xj}f_{xj}}{\sum_{j=1}^{m}s_{xj}}$. The net reproductive rate (*R*_0_), representing the expected number of offspring produced per individual, was calculated as: $R_{0}=\sum_{x=0}^{\infty }l_{x}m_{x}$. The intrinsic rate of increase (*r*) was estimated using the Lotka–Euler equation using age indexed from 0 as follows: $\sum_{x=1}^{\infty }e^{-r(x+1)}l_{x}m_{x}=1$. The finite rate of increase (*λ*) was determined as: $\lambda =e^{r}$. The mean generation time (*T*), which reflects the time required for a population to increase *R*_0_-fold under stable age-stage distribution, was computed as: $T=\frac{{lnR}_{0}}{r}$.

### 2.8. Data Analysis

All experimental data were organized in Microsoft Excel and analyzed using SPSS 23.0 (IBM Corp., Armonk, NY, USA). The corrected mortality rate was subjected to arcsine square root transformation before one-way analysis of variance (ANOVA) to assess treatment effects. Post hoc comparisons were performed using the LSD’s test, followed by correction of the comparison results with the Bonferroni-Holm method.

LC_50_ and LT_50_ were estimated by probit regression in SPSS 23.0, using dose–response and time–mortality regression models, respectively. Life history data for *T. absoluta* were analyzed within the age-stage, two-sex life table framework using TWOSEX-MSChart [30]. Developmental duration and reproductive parameters (oviposition and fecundity) between *B. bassiana*-colonized and control plants were compared via one-way ANOVA with LSD’s test, followed by correction of the comparison results with the Bonferroni-Holm method.

To ensure statistical robustness, 100,000 bootstrap resampling iterations were conducted [32], and a paired bootstrap test within TWOSEX-MSChart was used to compare population parameters (*R*_0_, *λ*, *r*, and *T*) between host plants.

## 3. Results

### 3.1. Identification of Strains

PCR amplification of the *B. bassiana* strains Bb1Bm, Bb2Bm, BbC, and BbM yielded fragment sizes of 598, 587, 573, and 575 bp, respectively. These sequences were submitted to the GenBank database for BLAST comparison.

**Figure 1:**
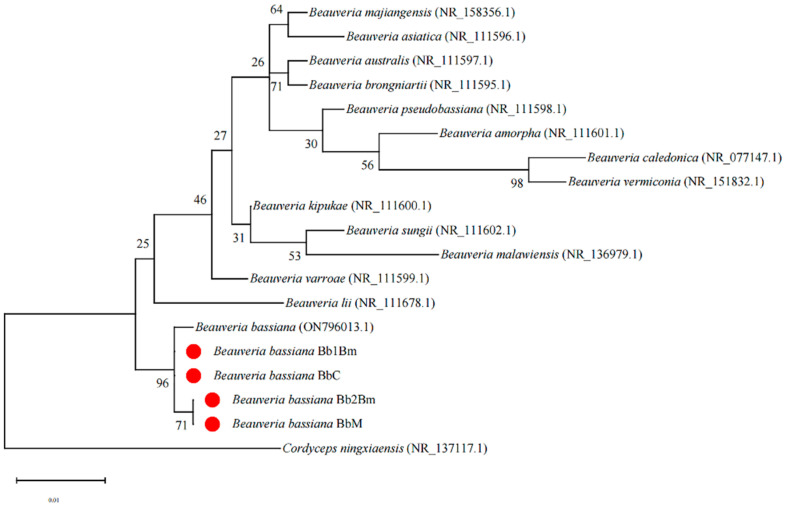
Phylogenetic tree of the *B. bassiana* strains Bb1Bm, Bb2Bm, BbC, and BbM, along with closely related strains, constructed using the Neighbor-Joining method based on ITS sequences. Numbers in parentheses indicate GenBank accession numbers. Bootstrap support values are displayed at each branch point. The scale bar represents a branch length of 0.01.

Phylogenetic analysis revealed that the four strains clustered within the same evolutionary branch as the *B. bassiana* strain (ON796013.1), exhibiting sequence similarities of 99.5%, 99.3%, 99.5%, and 98.6% for Bb1Bm, Bb2Bm, BbC, and BbM, respectively (Figure 1). Based on morphological characteristics and rDNA-ITS sequence similarity, the strains were conclusively identified as *B. bassiana*.

### 3.2. Evaluation of the Insecticidal Activity of B. bassiana Against Larvae of T. absoluta

All four *B. bassiana* strains displayed pathogenicity against second-instar *T. absoluta* larvae at the start of the experiment, though their virulence differed. Among them, Bb1Bm induced the highest larval mortality (57.0%), followed by BbM (55.0%), Bb2Bm (50.0%), and BbC (33.0%) when evaluated 14 days after exposure to 1 × 10^7^ conidia/mL. Despite these differences, larval mortality did not vary significantly among the four strains at this concentration (*F* = 1.957, *p* > 0.050) (Table 1).

The LT_50_ values were also determined (Table 1). BbM exhibited a lower LT_50_ (8.07 days), indicating the faster mortality induction, compared to Bb1Bm with an LT_50_ of 8.71 days. The cumulative mortality for Bb2Bm and BbC was insufficient to allow logistic regression fitting, preventing LT_50_ estimation for these strains.

### 3.3. Virulence of B. bassiana Bb1Bm Against Larvae of T. absoluta

The corrected mortality rate of second-instar *T. absoluta* larvae exhibited a dose-dependent response to increasing concentrations of *B. bassiana* Bb1Bm conidial suspensions. At the highest concentration (1 × 10^8^ spores/mL), larval mortality began to rise significantly from day 7 post-treatment, reaching 61.25% (*F* = 18.688, *p* < 0.001), which was significantly higher than at lower concentrations. By day 14, mortality further escalated to 85.00% (*F* = 16.124, *p* < 0.001), highlighting the strong virulence of Bb1Bm against *T. absoluta* larvae (Table 2). These results confirm a pronounced concentration-dependent mycocidal effect of *B. bassiana* Bb1Bm.

### 3.4. Colonization of B. bassiana in Tomato Plants

**Figure 2:**
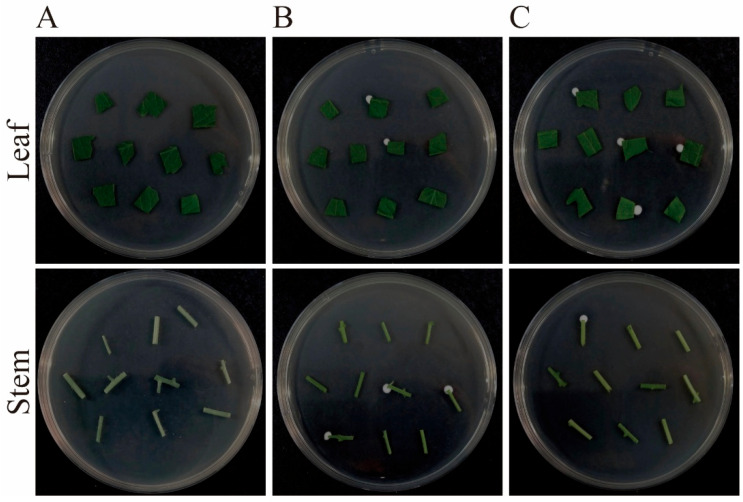
Detection of Colonization by *B. bassiana* in tomato plants: (**A**) Surface sterilization assessment following inoculation of tomato with *B. bassiana*. (**B**) Isolation and screening of endophytic fungi from tomato leaves and stems at 7 days post-inoculation with *B. bassiana* via foliar spray; (**C**) Isolation and screening of endophytic fungi from tomato leaves and stems at 7 days post-inoculation with *B. bassiana* via root irrigation.

**Figure 3:**
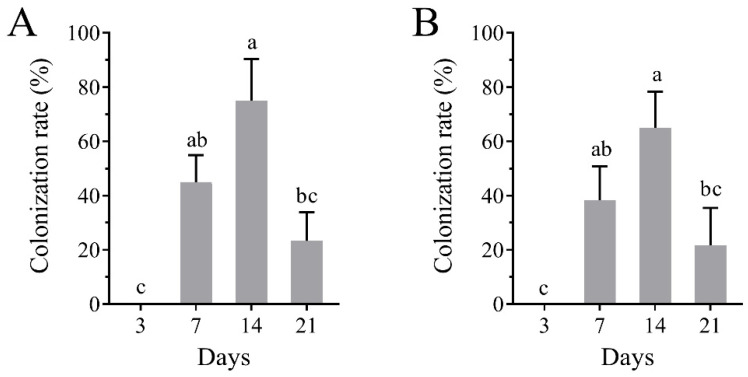
Colonization rate of *B. bassiana* in tomato. (**A**) BbFS; (**B**) BbRI. Different lowercase letters in the same column indicate significant differences (*p* < 0.05, LSD’s multiple range comparison).

Following surface sterilization, tomato leaves and stems were incubated on selective medium. As shown in Figure 2A, the sterilization protocol effectively eliminated surface microorganisms. After five days, the inoculated strain was successfully re-isolated from tomato leaf (Figure 2B) and stem tissues (Figure 2C), confirming the ability of *B. bassiana* to establish endophytic colonization within tomato plants. Colonization of Bb1Bm was detected in leaf tissues under both inoculation methods, becoming evident by day 7 post-inoculation (Figure 3). Colonization rates peaked at day 14, reaching 75.00% for foliar spraying and 65.00% for root irrigation. By day 21, the rates decreased to 23.33% and 21.67%, respectively, indicating a gradual decline in fungal persistence within host tissues (Figure 3).

### 3.5. Developmental Duration, Fecundity, and Offspring Sex Ratio of T. absoluta on Endophytic B. bassiana in Tomato

*T. absoluta* successfully completed its life cycle when reared on control tomato plants, as well as those treated with *B. bassiana* via foliar spraying (BbFS) and root irrigation (BbRI). Tomato cultivar significantly influenced pupal duration (*F* = 4.914, *p* < 0.010) and adult longevity for both sexes (female: *F* = 8.840, *p* < 0.010; male: *F* = 17.727, *p* < 0.010). BbFS and BbRI treatments significantly extended pupal development, reduced adult longevity, and markedly decreased female oviposition capacity (*F* = 6.540, *p* < 0.010). However, neither treatment had a significant effect on egg or larval duration (Table 3).

### 3.6. Life Table Analysis

**Figure 4:**
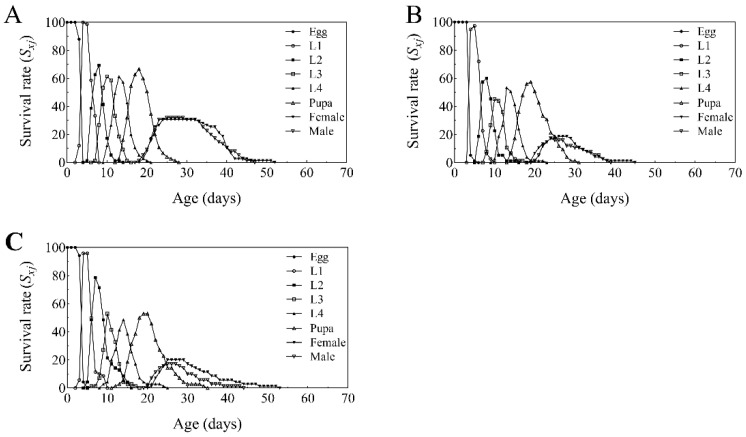
Specific age-stage survival rate (*S_xj_*) of *T. absoluta* that fed on control group tomato (**A**), BbFS (**B**), and BbRI (**C**).

The age-specific survival rate (*s_xj_*) of *T. absoluta* reared on tomato plants treated with BbFS and BbRI is shown in Figure 4. Overlapping survival curves indicate developmental asynchrony among individuals, resulting in distinct stage-specific overlaps. The probability of reaching adulthood was highest in the control group (62.67%, Figure 4A), whereas it dropped significantly in the BbFS (36.00%, Figure 4B) and BbRI (37.14%, Figure 4C) treatments.

**Figure 5:**
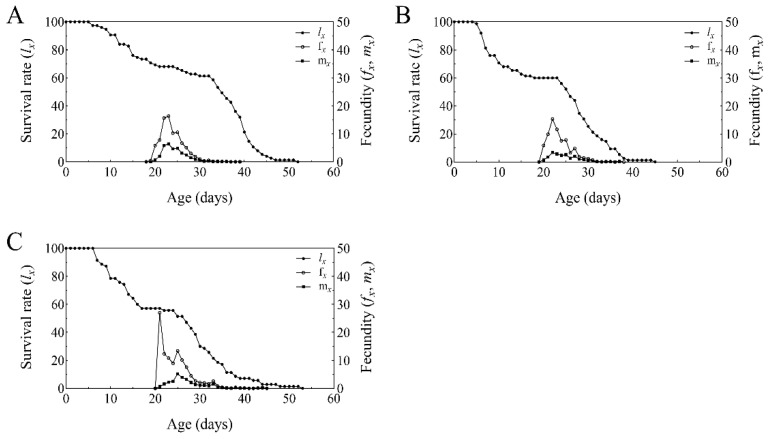
Specific age-stage survival rate (*l_x_*), specific age-stage fecundity (*f_x_*), and specific age-stage fecundity of total population (*m_x_*) of *T. absoluta* that fed on control group tomato (**A**), BbFS (**B**), and BbRI (**C**).

The age-stage-specific survival rate (*l_x_*), population age-stage-specific fecundity (*f_x_*), and age-stage-specific fertility (*m_x_*) curves are presented in Figure 5. The *l_x_* curve, a simplified representation of the *S_xj_* curve, does not account for individual variation. Compared to the control, the survival curves of *T. absoluta* under BbFS and BbRI treatments declined sharply between days 5–15, indicating higher mortality rates at larval stage (Figure 5).

The age-specific fecundity (*m_x_*) peaked on days 24, 23, and 25 in the control, BbFS, and BbRI treatments, respectively. The highest *mx* values recorded were 12.8, 6.8, and 10.3, respectively, while female fecundity onset was observed on days 20, 21, and 22 for the control, BbFS, and BbRI treatments, respectively (Figure 5).

**Figure 6:**
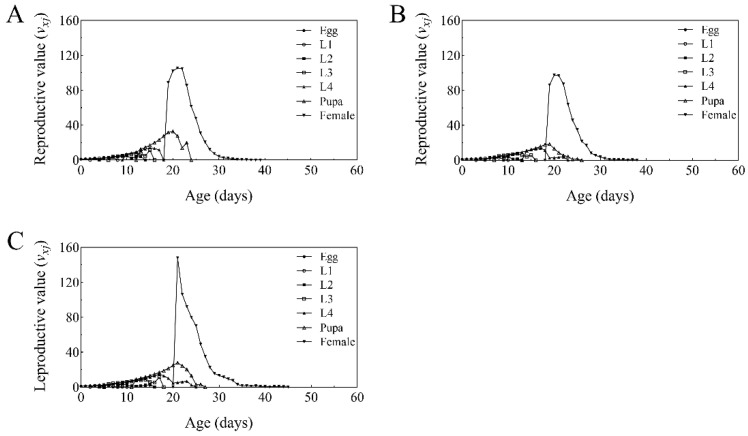
Specific age-stage reproductive value (*v_xj_*) of *T. absoluta* that fed on control group tomato (**A**), BbFS (**B**), and BbRI (**C**).

The age-stage-specific reproductive value (*v_xj_*) of *T. absoluta* increased significantly upon adult emergence-on day 19 in both the control and BbFS treatments, and on day 21 in the BbRI treatment. As adult lifespan increased, reproductive values gradually declined to zero on days 38, 38, and 44 for the control, BbFS, and BbRI treatments, respectively (Figure 6).

**Figure 7:**
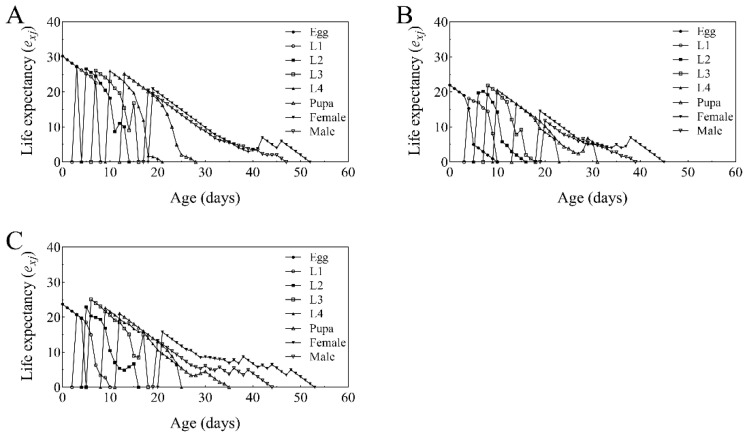
Specific age-stage life expectancies (*e_xj_*) of *T. absoluta* that fed on control group tomato (**A**), BbFS (**B**), and BbRI (**C**).

Age-stage life expectancy (*e_xj_*) represents the expected remaining lifespan of an individual at age *x* and stage *j*. As *x* increases, lifespan gradually declines to zero. The estimated life expectancy at hatching (*e_xj_*) was 30.23, 22.01, and 23.74 days for *T. absoluta* reared on control, BbFS, and BbRI-treated plants, respectively (Figure 7).

The control group exhibited significantly higher values (*p* < 0.05) for net reproductive rate (*R_0_*: 44.84 ± 7.93), finite rate of increase (λ: 1.16 ± 0.00), and intrinsic rate of increase (*r*: 0.15 ± 0.00) compared to both BbFS (*R_0_*: 20.53 ± 5.22; *λ*: 1.13 ± 0.00; *r*: 0.12 ± 0.01) and BbRI (*R_0_*: 25.95 ± 6.22; *λ*: 1.13 ± 0.01; *r*: 0.12 ± 0.00) treatments. However, no significant differences in *R_0_*, *λ*, or *r* were detected between BbFS and BbRI. Additionally, BbRI treatment resulted in a significantly longer mean generation time (*T*: 26.45 ± 0.51) than both the control (24.85 ± 0.26) and BbFS (24.59 ± 0.46), while no significant difference in *T* was observed between the control and BbFS (Table 4).

## 4. Discussion

*B. bassiana* is one of the earliest described entomopathogenic fungi and remains the most extensively studied and widely applied filamentous pathogen for insect control [33]. It combines broad environmental adaptability, scalable mass production methods, and strong biocontrol potential [33,34]. Its efficacy has been demonstrated against multiple lepidopteran pests, including *Spodoptera frugiperda* [35], *Helicoverpa armigera* [36], *Plutella xylostella* [37], and *Spodoptera exigua* [38]. In the present study, strain Bb1Bm showed high virulence against *T. absoluta* larvae, with a corrected mortality of 85.00% at 1 × 10^8^ conidia/mL, identifying it as a promising biocontrol candidate. Although this mortality is substantial, it is slightly lower than the >90% reported for strains LPP139, LEF140, and LEF141 at the same concentration [14], underscoring well-documented strain-specific differences in virulence. Moreover, strains achieving >90% mortality at lower concentrations have been reported (e.g., 1 × 10^7^ conidia/mL) [39], indicating that further strain screening or optimization of Bb1Bm application (e.g., formulation, timing) may improve efficacy.

Endophytic colonization of plants by *B. bassiana* can promote plant growth and confer antagonism to herbivores [40,41]. We achieved successful colonization of tomato via both foliar spray and root irrigation, with colonization becoming detectable at 7 days post-inoculation (dpi) and peaking at 14 dpi before declining. No significant difference in colonization efficiency was observed between inoculation methods (*p* > 0.05), a temporal pattern consistent with reports of maximal colonization at 14 dpi in tomato under varied inoculation regimes [42]. By contrast, some studies have observed progressive increases in root-inoculated plants reaching near-complete colonization at later time points [40]. Such discrepancies likely reflect strain-and host-specific interactions that govern colonization dynamics [43,44].

Feeding on *B. bassiana*-colonized tomato plants significantly reduced *T. absoluta* fitness, manifested as lower pre-adult survival, shortened adult longevity, and decreased fecundity relative to controls (*p* < 0.05). These effects are consistent with prior reports of reduced larval survival and increased mortality of *T. absoluta* on colonized tomatoes [14,45]. This extends to other herbivores. For example, impaired survival and reproduction of *Trialeurodes vaporariorum* nymphs on tomato [46] and reduced performance of *Tetranychus urticae* on bean [47]. Together, these studies indicate that endophyte-mediated suppression of herbivores is broadly effective across taxa.

Population life-table analyses corroborated the adverse demographic effects of endophytic colonization: net reproductive rate (*R*_0_), intrinsic rate of increase (*r*), and finite rate of increase (*λ*) were significantly reduced, while mean generation time (*T*) was prolonged (*p* < 0.05). These shifts indicate extended development and lower reproductive output on colonized plants, which together impose strong constraints on potential population growth of *T. absoluta*.

The significantly impaired fitness of *T. absoluta* on colonized tomatoes likely arises from two complementary plant-mediated mechanisms. First, endophytic colonization can induce or prime plant defense responses, elevating secondary metabolites and other anti-herbivore factors that indirectly reduce pest performance [48]. Examples include upregulation of *ZmWRKY36* in colonized maize, which suppresses *S. frugiperda* growth [49] and altered α-solanine levels in tomato that reduce *B. tabaci* reproduction [50]. Second, endophytes can secrete bioactive, insecticidal compounds within host tissues [51,52,53], with destruxin A from colonized tomatoes inducing lethality in *S. littoralis* [19].

## 5. Conclusions

In summary, this study identifies *B. bassiana* strain Bb1Bm as a highly virulent candidate for controlling *T. absoluta*. Life table analysis confirms that both foliar spray and root irrigation treatments significantly reduce *T. absoluta* population fitness, supporting the potential of *B. bassiana* as an effective component of integrated pest management strategies. However, the precise mechanisms through which *B. bassiana*-colonized tomato plants impair *T. absoluta* fitness, as well as the specific pathways involved, require further investigation.

## Figures and Tables

**Table 1:** Corrected mortality rates and probit regression parameters of four *B. bassiana* strains against *T. absoluta* larvae at 1 × 10^7^ spores/mL.

Strain	Corrected Mortality Rate (%)	Regression Equation	*R* ^2^	LT_50_ (Days)	95% Confidence Interval
Bb1Bm	57.00 ± 10.91 ^a^	Y = 58.781/(1 + Exp(3.142 − 0.560X))	0.956	8.71	8.74−10.70
Bb2Bm	50.00 ± 4.47 ^a^	Y = 49.349/(1 + Exp(3.677 − 0.719X))	0.995	—	—
BbM	55.00 ± 9.22 ^a^	Y = 56.994/(1 + Exp(3.749 − 0.708X))	0.977	0.977	7.21−8.94
BbC	33.00 ± 4.36 ^a^	Y = 36.042/(1 + Exp(3.731 − 0.508X))	0.938	—	—

Data in the “Corrected mortality rate (%)” column represent mean ± SE. Different lowercase letters in the “Corrected mortality rate (%)” column indicate significant differences within the same column (*p* < 0.05, LSD’s multiple range comparison). A dash “—“ indicates failure in fitting due to the relatively low mortality rate.

**Table 2:** Corrected mortality of the *B. bassiana* Bb1Bm strain at varying concentrations against *T. absoluta* larvae.

Spore Concentration (Spores/mL)	Corrected Mortality of Larvae at Different Time Points After Infection (%)		
	3 Days	7 Days	14 Days
1 × 10^8^	10.00 ± 3.78 ^a^	61.25 ± 5.32 ^a^	85.00 ± 4.43 ^a^
1 × 10^7^	7.50 ± 3.66 ^a^	34.38 ± 6.37 ^b^	57.50 ± 9.11 ^b^
1 × 10^6^	5.00 ± 3.27 ^a^	29.38 ± 4.36 ^b^	38.75 ± 5.32 ^b,c^
1 × 10^5^	5.00 ± 3.27 ^a^	21.25 ± 3.10 ^b,c^	33.75 ± 5.88 ^b,c^
1 × 10^4^	2.50 ± 2.50 ^a^	5.63 ± 3.71 ^c^	18.13 ± 6.19 ^c^

Data in the table represent mean ± SE. Different lowercase letters in the same column indicate significant differences (*p* < 0.05, LSD’s multiple range comparison).

**Table 3:** Duration of developmental stages and lifetime fecundity (mean ± SE) of *T. absoluta* on control group tomato, BbFS, and BbRI.

Parameters	Treatment		
	Control	BbFS	BbRI
Duration of egg (d)	3.89 ± 0.05 ^a^	3.96 ± 0.04 ^a^	3.96 ± 0.04 ^a^
Duration of 1st instar larval (d)	2.68 ± 0.10 ^a,b^	2.93 ± 0.13 ^a^	2.22 ± 0.08 ^b^
Duration of 2nd instar larval (d)	2.51 ± 0.10 ^b^	2.68 ± 0.12 ^b^	3.30 ± 0.13 ^a^
Duration of 3rd instar larval (d)	3.09 ± 0.15 ^a^	2.79 ± 0.13 ^a^	3.07 ± 0.16 ^a^
Duration of 4th instar larval (d)	3.32 ± 0.10 ^a^	3.68 ± 0.19 ^a^	3.52 ± 0.15 ^a^
Duration of Larva stage (d)	11.60 ± 0.18 ^a^	12.07 ± 0.21 ^a^	12.11 ± 0.26 ^a^
Duration of Pupa stage (d)	5.94 ± 0.16 ^b^	6.11 ± 0.25 ^a,b^	6.78 ± 0.19 ^a^
Adult female longevity (d)	18.48 ± 0.72 ^a^	12.20 ± 1.36 ^b^	13.27 ± 1.63 ^b^
Adult male longevity (d)	17.13 ± 0.86 ^a^	9.08 ± 1.20 ^b^	11.25 ± 1.19 ^b^
Number of eggs laid per female	147.82 ± 4.99 ^a^	112.07 ± 8.92 ^b^	124.79 ± 9.60 ^b^

Data are mean ± SE. Different lowercase letters in the same row indicate significant differences (*p* < 0.05, LSD’s multiple range comparison).

**Table 4:** Population parameters (mean ± SE) of *T. absoluta* fed on *B. bassiana*-colonized tomato and control group.

Parameters	Treatment		
	Control	BbFS	BbRI
Net reproductive rate (*R*_0_)	44.84 ± 7.93 ^a^	20.53 ± 5.22 ^b^	25.95 ± 6.22 ^b^
Finite rate of increase (*λ*)	1.16 ± 0.00 ^a^	1.13 ± 0.00 ^b^	1.13 ± 0.01 ^b^
Intrinsic rate of increase (*r*)	0.15 ± 0.00 ^a^	0.12 ± 0.01 ^b^	0.12 ± 0.00 ^b^
Mean generation time (*T*)	24.85 ± 0.26 ^b^	24.59 ± 0.46 ^b^	26.45 ± 0.51 ^a^

Data are mean ± SE. Different lowercase letters within the same row indicate significant differences (*p* < 0.05, LSD’s multiple range comparison).

## Data Availability

The original contributions presented in this study are included in the article. Further inquiries can be directed to the corresponding authors.
